# Purified and HPCCC Fractionated Anthocyanin-Rich Extracts from Black Bean Hulls Modulate H_2_O_2_-Induced Intracellular ROS Generation in RAW 264.7 Cells

**DOI:** 10.3390/molecules31193571

**Published:** 2026-10-08

**Authors:** Alejandro Escobedo, Sofía Barcena, Luis Mojica, Anne Gschaedler, Eugenia Lugo-Cervantes, Montserrat Alcazar

**Affiliations:** 1 School of Engineering and Sciences, Tecnológico de Monterrey, Monterrey 64700, Nuevo León, Mexico; a01648687@tec.mx; 2 Food Technology, Centro de Investigación y Asistencia en Tecnología y Diseño del Estado de Jalisco (CIATEJ), Camino Arenero 1227, Col. El Bajío, Zapopan 45019, Jalisco, Mexico; elugo@ciatej.mx; 3 Biotechnology Engineering, Instituto Tecnológico y de Estudios Superiores de Occidente, Anillo Periférico Sur Manuel Gómez Morín 8585, Col. Santa María Tequepexpan, Tlaquepaque 45604, Jalisco, Mexico; sofia.barcena@hotmail.com; 4 Industrial Biotechnology, Centro de Investigación y Asistencia en Tecnología y Diseño del Estado de Jalisco (CIATEJ), Camino Arenero 1227, Col. El Bajío, Zapopan 45019, Jalisco, Mexico; agschaedler@ciatej.mx

**Keywords:** natural pigment, agro-industrial by-product, black bean hulls, common bean, polyphenols, bioactive compounds

## Abstract

This study aimed to obtain crude, purified, and fractionated anthocyanin-rich extracts of three black bean cultivars from the South Pacific Region of Mexico (Chiapas, Guerrero, and Oaxaca). The extracts were obtained using an Amberlite XAD-7HP column and high-performance countercurrent chromatography (HPCCC), and their modulatory effect on oxidative stress was evaluated in RAW 264.7 macrophages. Purification significantly increased the concentrations of phenolic compounds and anthocyanins across all cultivars. However, HPCCC fractionation produced a cultivar-dependent response, with marked anthocyanin enrichment observed only in the Guerrero cultivar. In contrast, a decrease was noted for Oaxaca and Chiapas, compared with the corresponding purified extracts. The anthocyanin content per gram of extract ranged from 12.1 to 21.4 mg/g for crude extracts, 27.4 to 85.5 mg/g for purified extracts, and 48 to 98.8 mg/g for fractionated extracts. The purification and fractionation processes enabled the obtaining of anthocyanin-rich extracts from black bean hulls that reduced H_2_O_2_-induced intracellular ROS generation in a cultivar- and treatment-dependent manner, with the black bean cultivar from Chiapas being the most effective, indicating that differences in its phenolic composition may contribute to the observed biological response. These findings provide valuable insights into the potential of high-performance countercurrent chromatography for the fractionation of black bean anthocyanins.

## 1. Introduction

Anthocyanins are natural pigments that provide red, pink, blue, and purple colors in fruits, vegetables, and plants. They are a water-soluble subgroup of flavonoids with sugar moieties, including glucose, rutinose, galactose, and arabinose. The main anthocyanin aglycons (without sugar moieties) are cyanidin, delphinidin, peonidin, petunidin, malvidin, and pelargonidin [1]. Recently, anthocyanin applications have expanded to include natural pigments in the food, pharmaceutical, and cosmetic industries [2].

Black beans (*Phaseolus vulgaris* L.) are known to have a substantial concentration of anthocyanins, including delphinidin, petunidin, malvidin, and pelargonidin [3,4,5]. Black bean hulls are a by-product of the protein extraction process, and because of that, they are being recognized as a valuable source of anthocyanins [6]. Mexico has a wide range of common bean cultivars, including white, purple, red, yellow, brown, and black [7]. Small local farmers from the South Pacific Region of Mexico have carefully preserved endemic cultivars, resulting in distinct nutritional profiles [8]. Native black bean cultivars have been found to contain elevated concentrations of anthocyanins [9,10,11]. In addition to their pigment function, anthocyanins possess antioxidant and anti-inflammatory properties, which have been associated with potential health-promoting effects [12]. The antioxidant properties of anthocyanins found in other sources are widely recognized. Although the antioxidant activity of black bean anthocyanins has been explored using DPPH, ABTS, ORAC, FRAP, and radical-scavenging assays, and our previous studies have addressed their anti-inflammatory effects in cellular models, their specific role against H_2_O_2_-induced oxidative stress in macrophages has not been investigated [9,10,11,13]. Therefore, the novelty of this study relies on the combination of evaluating the H_2_O_2_-induced ROS model, comparing three endemic cultivars from the South Pacific Region of Mexico, valorizing their hulls as an agro-industrial by-product, and directly assessing how the degree of extract purification (crude, purified, and fractionated) impacts their efficacy in mitigating oxidative stress.

Anthocyanins are extracted from plant tissues using nonselective procedures. These crude extracts typically contain significant amounts of impurities, which we hypothesize may include sugars and organic acids that could potentially introduce bias in functional assays [14]. Adsorption purification methods using Amberlite XAD-7HP resin have successfully concentrated anthocyanins from several crude extracts. Further purification of the anthocyanin-rich extract can be achieved using high-performance countercurrent chromatography (HPCCC), an underutilized separation technique for anthocyanin fractionation [10,15]. HPCCC is a high-performance liquid–liquid partition chromatography version. This technique separates compounds based on partitioning between two immiscible liquids. The design of a solvent system that provides adequate separation is the primary challenge of this method [15].

This study aimed to obtain crude, purified, and fractionated anthocyanin-rich extracts from three black bean cultivars in the South Pacific Region of Mexico using an Amberlite XAD-7HP column and HPCCC, and to evaluate their modulatory effects on oxidative stress in RAW 264.7 macrophages.

## 2. Results

### 2.1. Extraction, Purification, and Fractionation

Black bean hulls must be separated from the cotyledon to extract anthocyanins efficiently. The beans are soaked to ensure complete hydration, facilitating dehulling. Once dried and dehulled, the hulls are milled to increase their interaction with the solvent system during extraction. Although the previous literature suggests that a small fraction of water-soluble anthocyanins is lost during soaking, this method is more effective than manual hull removal [16].

The percentage of bean hull obtained from whole beans appears in Table 1. These results are consistent with previously reported data [17]. Guerrero black beans had the lowest yield. We hypothesize this may be related to visually larger seeds, as larger seeds typically require longer soaking times to hydrate, allowing the hulls to separate fully.

Crude anthocyanin-rich extracts were obtained by conventional leaching; because the solvent system was not specific for anthocyanins, several compounds, including sugars and organic acids, were also extracted. The three crude extracts accounted for 9.2–11.4% of each hull’s weight. Regarding mass recovery during purification, higher percentages were observed, with recoveries ranging from 27.6–37.3% of the crude extract mass (Table 1). Adsorption resins allow retention of anthocyanins while removing most interfering compounds during elution. Among several adsorbents, Amberlite XAD-7HP has been shown to have the highest adsorption capacity for anthocyanins [18]. Purifying anthocyanin extracts with Amberlite XAD-7 HP removes almost all sugars, explaining the mass losses. In addition, the degradation constant and the non-enzymatic browning index of anthocyanins would decrease, as sugars are among the leading causes of anthocyanin degradation [19]. The extraction and purification yields of black bean anthocyanin-rich extracts were comparable to those reported by Carrera et al. [20]. The extraction yields ranged from 2.6 to 18.3% of the co-product weight. In comparison, the purification yield ranged from 0.8 to 5.6%.

**Figure 1:**
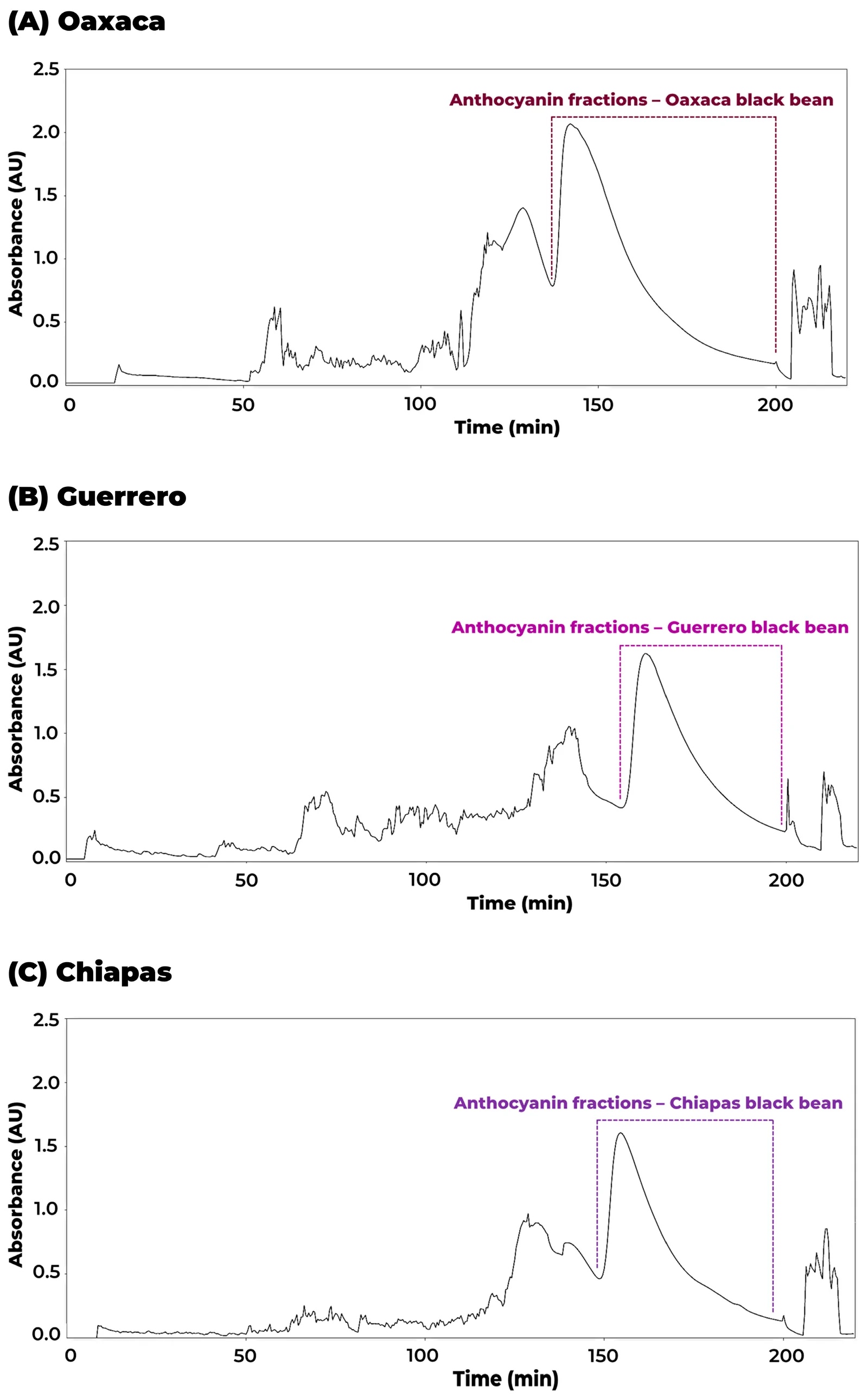
Representative HPCCC chromatogram of purified anthocyanin-rich extract of black beans.

The purified anthocyanin-rich extracts were then fractionated using HPCCC. The solvent system consisted of water, n-butanol, ethyl acetate, and acetic acid (5:4:1.5:0.5, *v*/*v*/*v*/*v*). It was selected based on the polarity, stability, and solubility of anthocyanins, since they are purified in strongly polar solvent systems. The partition coefficient (K value) fell within the acceptable range of 0.5 to 1.0 [21]. The anthocyanin fractions from black bean samples from Oaxaca, Guerrero, and Chiapas were collected within specific time ranges, as shown in Figure 1. The black bean Oaxaca sample was collected between 137 and 200 min, the black bean Guerrero sample between 155 and 198 min, and the black bean Chiapas sample between 135 and 198 min. Any samples collected before and after the collected anthocyanin fractions were discarded, as they represented uncharacterized fractions outside our target retention window.

The fractionated samples accounted for 19.3% to 22.2% of the purified extracts (Table 1). There have been few studies on the purification of anthocyanins from seeds using countercurrent chromatography. For example, cyanidin-3-O-β-D-glucoside was successfully concentrated from a crude black rice bran extract using HSCCC with the solvent system: water–n–butanol–tert–butyl–methyl–ether (TBME)–acetonitrile–trifluoroacetic acid (TFA) (5:2:2:1:0.1%) [22]. The weight yield of the cyanidin-rich extract was determined by the flow rate and ranged from 5.8 to 10.3% of the crude black rice bran extract. In this study, the yields of fractionated samples from crude anthocyanin-rich extracts ranged from 5.3 to 8.3%.

### 2.2. Total Phenolic Content

Table 2 shows the phenolic content in the anthocyanin-rich extracts obtained from Oaxaca, Guerrero, and Chiapas black bean cultivars. The crude anthocyanin-rich extracts obtained from Guerrero had a significantly higher phenolic content (275 mg GAE/g of extract) than those from Oaxaca and Chiapas, which presented similar phenolic contents (165 mg GAE/g of extract for both samples). This difference in phenolic content may be attributed to various factors, including genetics, soil composition, harvesting year, and fertilizer use [14].

The purification process led to a significant phenolic enrichment (*p* < 0.05) across all regions. The purified extracts from the black bean Chiapas sample showed more than a threefold increase in phenolic content. After purification, the black bean Guerrero extract had a similar content of phenolic compounds to the other purified extracts, even though the black bean crude extract from Guerrero had a higher phenolic content. One possible explanation could be differences in the non-phenolic matrix composition, which could result in a lower relative concentration of phenolic compounds during purification. However, we did not determine the sugar content in the present study [18]. Additionally, Guerrero’s black bean crude extract may have a different natural phenolic profile that favors phenolic elution during purification.

As expected, the fractionated extracts from the three regions showed the highest phenolic content among all the samples. This enrichment may reflect the removal of non-phenolic matrix components during HPCCC fractionation. These outcomes are consistent with previous studies demonstrating that purification techniques can enhance the total phenolic content of black bean anthocyanin-rich extracts [10,13].

### 2.3. Anthocyanin Quantification by UHPLC

The concentrations of the anthocyanins within the extracts are shown in Table 3. Meanwhile, Figures S1–S3 show delphinidin-3-O-glucoside, petunidin-3-O-glucoside, and malvidin-3-O-glucoside in the UHPLC chromatograms, representing the three anthocyanins isolated from the anthocyanin-rich extracts. Across all stages and regions, delphinidin-3-O-glucoside was the most abundant anthocyanin. Previous studies have found these three anthocyanins in similar proportions in black beans [4,5]. Among the crude anthocyanin-rich extracts, Guerrero black bean extract had the highest (*p* < 0.05) concentration of delphinidin-3-O-glucoside, petunidin-3-O-glucoside, and malvidin-3-O-glucoside. This suggests a higher initial anthocyanin content than other regions, similar to the total phenolic content [23].

After purification with Amberlite XAD-7HP resin, extracts from all three regions increased anthocyanin content (*p* < 0.05), with the Oaxaca black bean extract showing a significant increase, particularly for delphinidin-3-O-glucoside (41.1 mg/g). Similar proportions among the three anthocyanins were maintained, and their molecular structure might not have been affected during this process, as previously reported [19]. However, the purified black bean extract from Guerrero had the lowest anthocyanin content (*p* < 0.05), despite having the highest content before purification. As with phenolic compounds, this suggests that Guerrero’s crude black bean extract might have different adsorption/desorption behaviors on the resin, distinct matrix effects, or differences in analytical recovery that limit anthocyanin enrichment during purification. In a similar study, Fonseca-Hernandez et al. [13] obtained a phenolic-enriched extract from the seed hulls of native Mexican black beans via conventional leaching, followed by purification using Amberlite XAD-7HP resins. The extract contained 230 mg of delphinidin-3-O-glucoside, 149 mg of malvidin-3-O-glucoside, and 38 mg of petunidin-3-O-glucoside per gram of extract. This high concentration of anthocyanins could be attributed to manual hull removal, which helped prevent anthocyanin loss during soaking. According to Alcázar-Valle et al. [9], some native Mexican black bean cultivars contain more anthocyanins naturally than those used in this study (Table 4). Specific cultivars could contain up to 9.42 mg of cyanidin-3-O-glucoside equivalents (C3GE) per g of seed coat.

The fractionation resulted in the Guerrero black bean extract achieving the highest concentrations of delphinidin-3-O-glucoside, petunidin-3-O-glucoside, and malvidin-3-O-glucoside. In contrast, the content of all three anthocyanins decreased in the Oaxaca and Chiapas black bean extracts. This clear cultivar-dependent response to HPCCC fractionation, in which varieties from Oaxaca and Chiapas show enrichment after Amberlite purification but a decrease after HPCCC, whereas the Guerrero variety exhibits the opposite behavior, demonstrates that processing efficiency depends largely on the matrix. The black bean Guerrero sample had the lowest concentration before fractionation but the highest afterward. This can be observed in the HPCCC chromatograms (Figure 1), where the Guerrero black bean extract showed better peak resolution than the other samples.

For future studies, each anthocyanin could be isolated by employing a second run of high-performance countercurrent chromatography (HPCCC). This procedure is commonly used for co-eluted compounds and has already been implemented to isolate anthocyanins through HPCCC; Mbeunkui et al. [24] collected four significant fractions from a crude blackcurrant extract using HPCCC with a two-phase solvent system consisting of tert-butyl-methyl-ether (TBME)–butanol–acetonitrile–water (1:4:1:5 + 0.1% TFA). The first fraction was then resubmitted to HPCCC using the same solvent system, with the flow rate reduced from 20 mL/min to 10 mL/min. Finally, they successfully isolated each of the four main anthocyanins in blackcurrant: delphinidin-3-O-rutinoside, cyanidin-3-O-rutinoside, delphinidin-3-O-glucoside, and cyanidin-3-O-glucoside.

Table 4 illustrates the total anthocyanin content related to anthocyanin-rich extract, bean hull, and whole bean. The total anthocyanin content per gram of extract ranged from 12.1 to 21.4 mg/g for crude extracts, 27.4 to 85.5 mg/g for purified extracts, and 48 to 98.8 mg/g for fractionated extracts. These values are higher than those of extracts obtained from other innovative by-products. For instance, Carrera et al. [20] obtained crude extracts from cob, leaves, and grains of Colombian purple corn, with total anthocyanin concentrations of 0.64 mg/g, 0.37 mg/g, and 0.24 mg/g, respectively. They then purified these extracts using Amberlite XAD-7HP. After purification, the total anthocyanin concentration increased to 1.74 mg/g in the cob, 0.46 mg/g in the leaves, and 0.42 mg/g in the grains. Moreover, conventional leaching was an efficient and practical method for obtaining crude extracts with anthocyanin concentrations comparable to those from alternative extraction methods, such as microwave-assisted extraction. In a study by Mali and Kumar [25], the total anthocyanin content of the extracts ranged from 3.4 to 40.5 mg/g dry matter.

It is noteworthy that the apparent recovery of total anthocyanins after purification exceeded 100% for Oaxaca and Chiapas when expressed per 100 g of bean hull. This phenomenon reflects strong matrix effects in the crude extracts, such as co-eluting macromolecules that might inhibit analytical detection, which are removed after Amberlite purification, rather than an actual generation of new anthocyanin mass. Conversely, apparent recovery following HPCCC fractionation dropped significantly for Oaxaca and Chiapas compared to Guerrero. This highlights an inverse correlation between purity and total yield. While HPCCC can concentrate specific anthocyanins or remove unwanted matrix, the loss of total analyte mass in certain cultivars requires careful evaluation regarding potential industrial applications.

On the other hand, a crude extract containing 137.9–239.5 mg of total anthocyanins can be produced from 100 g of native Mexican black bean hulls. Similar concentrations can be obtained using other alternative extraction methods. Hsieh-Lo et al. [4] optimized the extraction of black bean anthocyanin-rich extracts using supercritical fluid extraction (SFE) and pressurized liquid extraction (PLE). The optimal conditions for both extraction methods yielded extracts containing 226 mg of C3GE per 100 g of bean hull for PLE and 264 mg for SFE. These findings demonstrate that black bean hulls are a rich, untapped source of anthocyanins. In comparison, this co-product can produce crude extracts with anthocyanin contents similar to those of blueberries, one of the most popular sources of anthocyanins [26].

### 2.4. Antioxidant Effect of Anthocyanin-Rich Extracts

RAW 264.7 macrophages were pre-treated with anthocyanin-rich extracts and stimulated with 1 mM hydrogen peroxide (H_2_O_2_) to assess cell viability under oxidative stress. Cells were treated with different concentrations of extracts from 2.5 to 10 μg of extract/mL. Since concentrations of 5 and 10 μg/mL exhibited mild cytotoxicity in some fractionated samples, 2.5 μg/mL was selected as the highest safe concentration across all treatments to ensure a fair comparison (Figure S4). Then, the protective effect of the anthocyanin-rich extracts on reactive oxygen species (ROS) generation was evaluated using the DCF-DA fluorescence assay.

**Figure 2:**
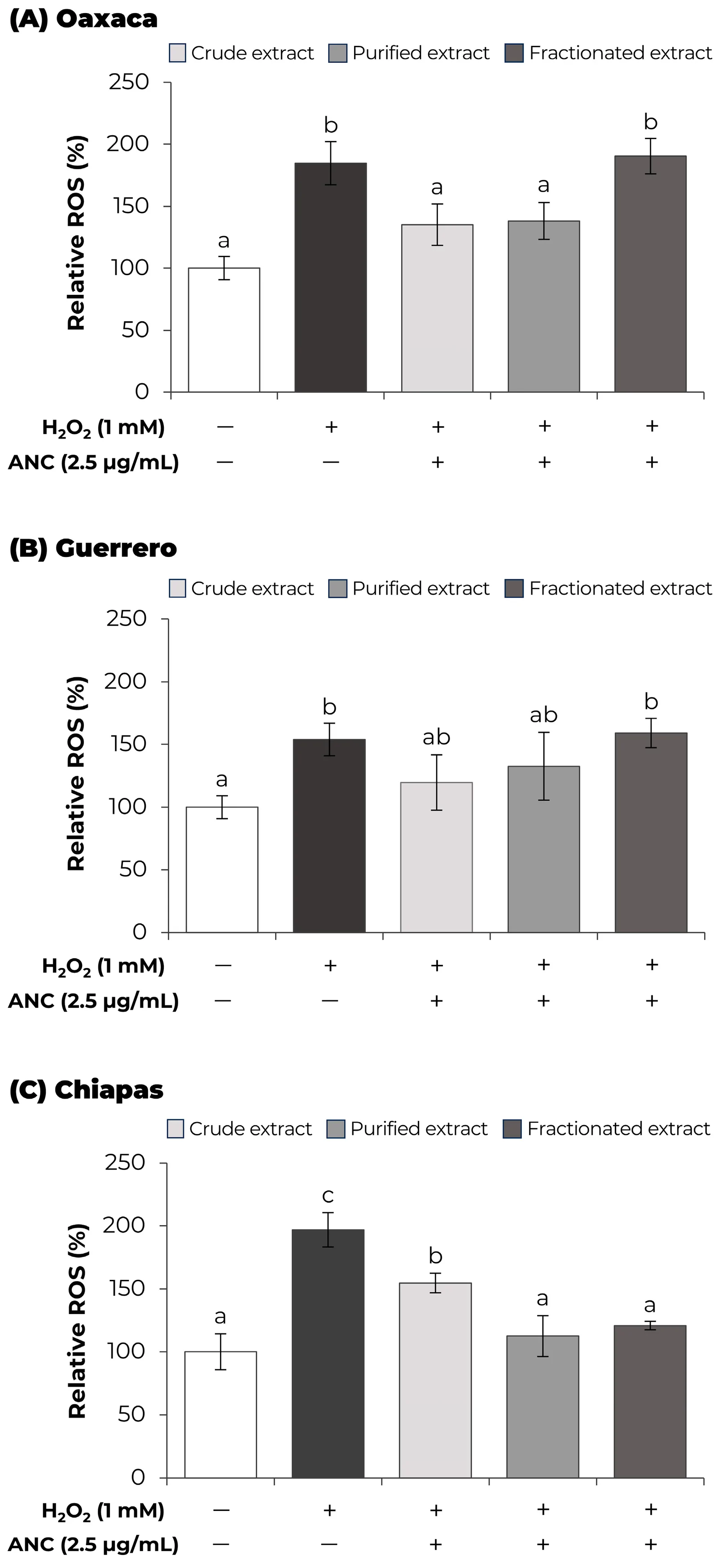
Effect of anthocyanin-rich extracts on ROS generation in RAW 264.7 macrophages stimulated with 1 mM hydrogen peroxide (H_2_O_2_). The five bars represent: (1) Control (untreated cells); (2) H_2_O_2_ (oxidative stress control); (3) Crude extract + H_2_O_2_; (4) Purified extract + H_2_O_2_; and (5) Fractionated extract + H_2_O_2_. Mean values ± SD. Values with different letters indicate significant differences (*p* < 0.05) among treatments using ANOVA and Tukey’s post hoc test.

As shown in Figure 2, the stimulation with 1 mM H_2_O_2_ for 30 min resulted in a significant increase in DCF-DA fluorescence intensity compared with the control. The relative percentage reduction was calculated as (H_2_O_2_ − sample)/H_2_O_2_ × 100. For black bean Oaxaca samples, the pre-treatment with 2.5 μg/mL of crude and purified extracts significantly decreased the ROS production by 27% and 25%, respectively (Figure 2A). Meanwhile, the fractionated extract did not alter ROS levels. Interestingly, the reductions in fluorescence intensity were not significant for the Guerrero extracts, suggesting that future investigations could explore dose–response experiments if non-cytotoxic higher concentrations can be achieved (Figure 2B). All three anthocyanin-rich extracts from Chiapas black beans reduced ROS generation by 21% for the crude extract, 43% for the purified extract, and 39% for the fractionated extract (Figure 2C).

The results showed that anthocyanin-rich extracts from black beans exhibited antioxidant potential. This anthocyanin property has been extensively studied, demonstrating that each has different antioxidant activity [27]. Delphinidin-3-O-glucoside is the major anthocyanin found in these black bean extracts. It has three hydroxyl groups (-OH) on the B-ring, which can be oxidized by donation of one electron (semiquinone form) or two electrons (quinone form), thereby conferring antioxidant activity against free radicals [28].

The biological results did not demonstrate a direct linear relationship between anthocyanin concentration and the reduction in intracellular ROS. Guerrero fractionated extracts had the highest anthocyanin concentrations but showed little ROS reduction, whereas Chiapas extracts showed strong cellular responses despite lower concentrations. This suggests that anthocyanin enrichment alone does not determine biological efficacy. Other unidentified components, as well as synergistic or antagonistic interactions within the matrix, may play a significant role [29,30]. Conversely, as observed by Fonseca-Hernández et al. [13], the purification of black bean extracts increased the total phenolic content, which showed a strong negative correlation with the half-maximal inhibitory potential (IC50) of antioxidant activity (r = −0.93) and radical-scavenging potential (r = −0.95).

It should be noted as a limitation of this study that specific controls for extract autofluorescence or optical quenching were not performed, which could introduce minor optical interferences in the DCF-DA assay given the pigmented nature of the extracts. Furthermore, the absence of a positive antioxidant reference control limits the absolute validation of the cellular model’s sensitivity.

## 3. Materials and Methods

### 3.1. Materials

Three black bean (*P. vulgaris*) cultivars were obtained from rural communities in the South Pacific Region of Mexico between July and September 2019 (Table 5) and stored at –18 °C for approximately 2 years prior to extraction and analysis. RAW 264.7 macrophages (TIB-71) were purchased from the American Type Culture Collection (Manassas, VA, USA), and Dulbecco’s Modified Eagle’s Medium (DMEM) (10-013-CM) was acquired from Corning (Corning, NY, USA). Fetal bovine serum (FBS) was obtained from SF-FB, BY PRODUCTOS (Guadalajara, Mexico), and penicillin–streptomycin (1107444000) from F. Hoffmann-La Roche Ltd. (Basel, Switzerland). Malvidin-3-O-glucoside (M3G) (89728), petunidin-3-O-glucoside (P3G) (89755), and delphinidin-3-O-glucoside (D3G) (89627) standards were purchased from PhytoLab GmbH & Co. KG (Vestenbergsgreuth, Germany). All other reagents were obtained from Sigma-Aldrich (St. Louis, MO, USA) unless otherwise specified.

### 3.2. Dehulling, Extraction, and Purification Process

The dehulling process consisted of soaking the black beans in distilled water (1:1.2, *w*/*v*) at 40 °C for 4 h. After soaking, the beans were dried in a convection oven at 55 °C for 6 h. Then, a dehulling machine (Guadalajara, Mexico) was used to separate the hull from the cotyledon. The hulls were milled and sieved through a 0.59 mm mesh, and then mixed with ethanol/water/formic acid (70:29.7:0.3, *v*/*v*/*v*) at a 1:25 (*w*/*v*) ratio and stirred at 40 °C for 24 h. The mixtures were then filtered, concentrated using a rotary evaporator at 40 °C (R-100, Büchi, Flawil, Switzerland), and freeze-dried. Anthocyanin-rich extracts were resuspended with ethanol/water/formic acid (70:29.7:0.3, *v*/*v*/*v*) to 50 mg/mL and filtered through a 0.45 µm nylon membrane. Next, 10 mL of the resulting mixture was loaded onto an Amberlite XAD-7HP column (45.7 × 2.54 cm) (Sigma-Aldrich) at a flow rate of 1 BV/h. The column was rinsed with a 0.3% formic acid solution, and retained compounds were eluted with ethanol/water/formic acid (70:29.7:0.3, *v*/*v*/*v*) at 4 BV/h. The purified anthocyanin-rich extract was concentrated, freeze-dried, and stored at −18 °C until further processing.

### 3.3. HPCCC Procedure

The Spectrum HPCCC (Dynamic Extractions Ltd., Blaenau Gwent, UK) was used to obtain anthocyanin fractions using a two-phase solvent system of water/n-butanol/ethyl acetate/acetic acid (5:4:1.5:0.5, *v*/*v*/*v*/*v*). A 200 mg sample of the purified anthocyanin-rich extract was dissolved in 6 mL of the upper phase/lower phase (1:1, *v*/*v*) and filtered through a 0.2 µm nylon membrane. The elution-extrusion method was performed in reverse mode. The semi-preparative column (136 mL) was maintained at 1600 rpm and 25 °C. The mobile phase was pumped at a flow rate of 0.75 mL/min for 200 min during the elution stage. Meanwhile, at extrusion, the stationary phase was pumped at 9 mL/min for 20 min. The effluent was monitored using a Flash 14 DAD detector (ECOM spol. s.r.o., Chrášťany u Prahy, Czech Republic) at 520 nm. The fractions were then concentrated using a centrifugal vacuum concentrator at 1000× *g* and 40 °C, freeze-dried, and stored at −18 °C until analysis.

### 3.4. Total Phenolic Content Analysis

Total polyphenols were analyzed using an adaptation of the Folin–Ciocalteu assay [4]. Anthocyanin-rich extracts were reconstituted in ethanol/water/formic acid (70:29.7:0.3, *v*/*v*/*v*) and diluted in distilled water (1:100, *v*/*v*). Then, 50 µL was added to a 96-well flat-bottom plate along with 50 µL of the Folin–Ciocalteu reagent. The mixture was allowed to stand for five minutes. Finally, 100 µL of a 20% sodium carbonate solution (*w*/*v*) was added, and the mixture was incubated for an additional 10 min. The absorbance was read at 765 nm (Infinite M200 Pro, TECAN, Männedorf, Switzerland), and the results were expressed as gallic acid equivalents (GAE) using a calibration curve (25–150 µg/mL, R^2^ = 0.99) obtained from independent extraction replicates.

### 3.5. Anthocyanin Quantification by UHPLC

Anthocyanin quantification was performed using a UHPLC method [9]. Freeze-dried anthocyanin-rich extracts were resuspended with ethanol/water/formic acid (70:29.7:0.3, *v*/*v*/*v*) and filtered through a 0.2 µm nylon membrane. A UHPLC system equipped with a PDA detector (Acquity Arc, Waters Corporation, Milford, MA, USA) and a CORTECS C18 Column (2.7 µm, 4.6 mm × 150 mm) (186007378, Waters Corporation, Milford, MA, USA) was used to analyze the anthocyanin-rich extracts at a wavelength of 520 nm. The injection volume was 20 μL, and the flow rate was 0.5 mL/min. The solvent system comprised acidified water with 0.1% trifluoroacetic acid (mobile phase A) and acetonitrile (mobile phase B). Gradient conditions were as follows: 0 min: 10% B; 12 min: 18% B; 20 min: 28% B; 21 min: 40% B; 23 min: 40% B; 25 min: 10% B; and 30 min: 10% B. Malvidin 3-O-glucoside (R^2^ = 0.999), petunidin 3-O-glucoside (R^2^ = 0.999), and delphinidin 3-O-glucoside (R^2^ = 0.996) were quantified using a calibration curve with 6 points ranging from 0.5–20 μg/mL.

### 3.6. Cell Culture and Viability

RAW 264.7 macrophages were cultured in DMEM, containing 10% (*v*/*v*) FBS and 1% (*v*/*v*) penicillin–streptomycin at 37 °C in a humidified atmosphere with 5% CO_2_. They were seeded at a density of 104 viable cells per well in a 96-well plate and incubated for 24 h. Macrophages were pre-treated with crude, purified, and fractionated extracts (10, 5, 2.5 μg/mL) resuspended in serum-free DMEM. After 1 h of pre-treatment, cells were stimulated with 1 mM hydrogen peroxide (H_2_O_2_) in serum-free DMEM for 30 min and washed twice with PBS. AlamarBlueTM (10% *v*/*v* in complete growth medium) was added to each well, and the plate was incubated for two hours. Fluorescence was measured using an excitation wavelength of 560 nm and an emission of 590 nm. Cell viability was calculated relative to untreated control cells.

### 3.7. Reactive Oxygen Species Assay of Anthocyanin-Rich Extracts

The reactive oxygen species (ROS) assay was carried out with minor modifications [31]. Intracellular ROS generation was determined using the 2′,7′-dichlorofluorescein diacetate (DCF-DA) method. Macrophages were seeded into a 96-well plate at a density of 10^4^ viable cells per well and incubated for 24 h. Then, 20 μM DCF-DA in serum-free DMEM was added, and the mixture was incubated for 20 min. They were then pre-treated with crude, purified, and fractionated extracts at 2.5 μg/mL resuspended in serum-free DMEM for 1 h. This sequence was modified to allow cells to internalize the probe before treatment, preventing direct optical interaction between the pigmented extracts and the probe in the extracellular medium. After that, cells were stimulated with 1 mM H_2_O_2_ in serum-free DMEM. After 30 min of incubation, they were washed twice with PBS, and fluorescence was read with excitation and emission wavelengths of 485 and 530 nm, respectively.

### 3.8. Statistical Analysis

Values are expressed as the mean ± SD from two independent extraction batches, with three technical replicates analyzed per batch. Technical replicates were averaged within each independent biological replicate prior to statistical analysis. Means were analyzed by one-way ANOVA, followed by Tukey’s test with a significance level of *p* < 0.05. Statistical analyses were performed using Statgraphics Centurion 19 version 19.6.02 (Statistical Graphics Corp., The Plains, VA, USA).

## 4. Conclusions

Purification using Amberlite XAD-7HP and fractionation through HPCCC significantly boosted the concentration (enrichment) of total phenolic compounds and three specific anthocyanins for specific cultivars, though total apparent recovery varied among cultivars and processing stages. The cellular response varied according to cultivar and processing stage, and did not directly parallel anthocyanin concentration. The purified extracts showed better biological efficacy, while HPCCC fractionation did not further improve it.

The Chiapas black bean hull extracts showed the strongest ability to reduce H_2_O_2_-induced intracellular ROS generation in RAW 264.7 macrophages. While these findings offer valuable insights into the potential of anthocyanin-rich black bean extracts, further in vivo studies are required before considering potential health-related applications. Finally, the results of this study are expected to help characterize and valorize native black bean varieties and contribute to their preservation in the development of the South Pacific Region of Mexico.

## Figures and Tables

**Table 1:** Percentage yields of dehulling, extraction, and purification/fractionation mass recoveries.

Sample	Oaxaca	Guerrero	Chiapas
Hull yield ^1^	7.0%	5.8%	7.8%
Crude anthocyanin-rich extract yields ^2^	9.2%	11.2%	11.4%
Purified anthocyanin-rich extract recovery ^3^	27.6%	37.3%	27.6%
Fractionated anthocyanin-rich extract recovery ^4^	20.6%	22.2%	19.3%

^1^ Hull obtained from whole beans. ^2^ Crude anthocyanin-rich extract obtained from black bean hulls. ^3^ Purified anthocyanin-rich extract obtained from the crude anthocyanin-rich extract mass. ^4^ Fractionated anthocyanin-rich extract obtained from purified anthocyanin-rich extract mass.

**Table 2:** Total phenolic content in crude, purified, and fractionated anthocyanin-rich extracts.

Sample	Oaxaca mg GAE/g of Extract	Guerrero mg GAE/g of Extract	Chiapas mg GAE/g of Extract
Crude anthocyanin-rich extract	165 ± 14 ^bC^	275 ± 16 ^aC^	165 ± 14 ^bC^
Purified anthocyanin-rich extract	552 ± 26 ^aB^	491 ± 32 ^aB^	589 ± 63 ^aB^
Fractionated anthocyanin-rich extract	681 ± 31 ^aA^	624 ± 18 ^aA^	701 ± 42 ^aA^

Mean values ± SD. Values within a row with different lowercase letters indicate significant differences (*p* < 0.05) among cultivars with the same treatment. In contrast, values within a column with different uppercase letters indicate significant differences (*p* < 0.05) between treatments using ANOVA and Tukey’s post hoc test. GAE, gallic acid equivalents.

**Table 3:** Anthocyanin content in crude, purified, and fractionated anthocyanin-rich extracts by UHPLC (mg/g of extract).

Treatment	Oaxaca	Guerrero	Chiapas
*Crude anthocyanin-rich extract*			
Delphinidin-3-O-G	8.3 ± 0.1 ^bC^	11.4 ± 0.3 ^aC^	6.8 ± 0.1 ^cC^
Petunidin-3-O-G	5.7 ± 0.3 ^aC^	6.4 ± 0.1 ^aC^	3.6 ± 0.1 ^bC^
Malvidin-3-O-G	3.2 ± 0.2 ^aC^	3.6 ± 0.4 ^aC^	1.7 ± 0.03 ^bC^
*Purified anthocyanin-rich extract*			
Delphinidin-3-O-G	41.1 ± 1.7 ^aA^	14.4 ± 0.02 ^cB^	36.7 ± 0.7 ^bA^
Petunidin-3-O-G	29.2 ± 0.4 ^aA^	8.0 ± 0.5 ^cB^	20.8 ± 1.1 ^bA^
Malvidin-3-O-G	15.2 ± 0.4 ^aA^	5.0 ± 0.1 ^bB^	12.6 ± 1.8 ^aA^
*Fractionated anthocyanin-rich extract*			
Delphinidin-3-O-G	30.5 ± 0.7 ^bB^	55.5 ± 0.8 ^aA^	28.1 ± 0.3 ^bB^
Petunidin-3-O-G	18.5 ± 0.2 ^bB^	31.0 ± 2.6 ^aA^	15.2 ± 0.4 ^bB^
Malvidin-3-O-G	7.6 ± 0.9 ^bB^	12.3 ± 0.7 ^aA^	4.7 ± 0.1 ^cB^

Mean values ± SD. Values within a row with different lowercase letters indicate significant differences (*p* < 0.05) among cultivars with the same treatment. In contrast, values within a column with different uppercase letters indicate significant differences (*p* < 0.05) between treatments of the same anthocyanin using ANOVA and Tukey’s post hoc test.

**Table 4:** Total anthocyanin content in anthocyanin-rich extracts, bean hull, and whole bean by UHPLC.

Treatment	Oaxaca	Guerrero	Chiapas
*mg of total anthocyanins/g of extract*			
Crude anthocyanin-rich extract	17.1 ± 0.1 ^b^	21.4 ± 0.6 ^a^	12.1 ± 0.1 ^c^
Purified anthocyanin-rich extract	85.5 ± 2.4 ^a^	27.4 ± 0.6 ^c^	70.1 ± 3.6 ^b^
Fractionated anthocyanin-rich extract	56.5 ± 0.1 ^b^	98.8 ± 2.7 ^a^	48.1 ± 0.6 ^c^
*mg of total anthocyanins/100 g of bean hull*			
Crude anthocyanin-rich extract	157.5 ± 0.5 ^b^	239.5 ± 6.6 ^a^	137.9 ± 0.3 ^c^
Purified anthocyanin-rich extract	217.2 ± 6.1 ^a^	114.3 ± 2.5 ^b^	220.5 ± 11.3 ^a^
Fractionated anthocyanin-rich extract	29.6 ± 0.1 ^b^	91.6 ± 2.5 ^a^	29.2 ± 0.4 ^b^
*mg of total anthocyanins/100 g of whole bean*			
Crude anthocyanin-rich extract	11.0 ± 0.1 ^b^	13.9 ± 0.4 ^a^	10.8 ± 0.1 ^b^
Purified anthocyanin-rich extract	15.2 ± 0.4 ^b^	6.6 ± 0.1 ^c^	17.2 ± 0.9 ^a^
Fractionated anthocyanin-rich extract	2.1 ± 0.1 ^b^	5.3 ± 0.1 ^a^	2.3 ± 0.1 ^b^

Mean values ± SD. Values within a row with different lowercase letters indicate significant differences (*p* < 0.05) among cultivars with the same treatment using ANOVA and Tukey’s post hoc test.

**Table 5:** Black bean cultivars from the South Pacific Region of Mexico.

Region	Common Name	Image
Chiapas	*Negro cubano*	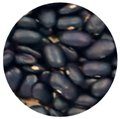
Guerrero	*Negro de mata*	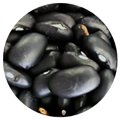
Oaxaca	*Criollo Tiltepec*	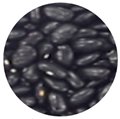

## Data Availability

The raw data supporting the conclusions of this article will be made available by the authors on request.

## Supplementary Materials

The following supporting information can be downloaded at: https://www.mdpi.com/article/10.3390/molecules31193571/s1, Figure S1: HPLC chromatogram of anthocyanin extracts from Oaxaca black beans; Figure S2: HPLC chromatogram of anthocyanin extracts from Guerrero black beans; Figure S3: HPLC chromatogram of anthocyanin extracts from Chiapas black beans; Figure S4: Effect of anthocyanin extracts on cell viability in RAW 264.7 macrophages stimulated with 1 mM hydrogen peroxide (H_2_O_2_).

## Abbreviations

The following abbreviations are used in this manuscript:

D3G	Delphinidin 3-O-glucoside
DCF-DA	Dichlorofluorescein diacetate
DMEM	Dulbecco’s Modified Eagle’s Medium
FBS	Fetal bovine serum
GAE	Gallic acid equivalents
HPCCC	High-performance countercurrent chromatography
HSCCC	High-speed countercurrent chromatography
M3G	Malvidin 3-O-glucoside
P3G	Petunidin 3-O-glucoside
PBS	Phosphate-buffered saline
