# Supplementary material for: Purified and HPCCC Fractionated Anthocyanin-Rich Extracts from Black Bean Hulls Modulate H2O2-Induced Intracellular ROS Generation in RAW 264.7 Cells
Source: Molecules. 2026 Oct 8;31(19):3571. doi: 10.3390/molecules31193571 (PMC13667101; doi:10.3390/molecules31193571)
Supplement: Supplementary file 1 [file molecules-31-03571-s001.zip › molecules-4557238-supplementary.pdf]

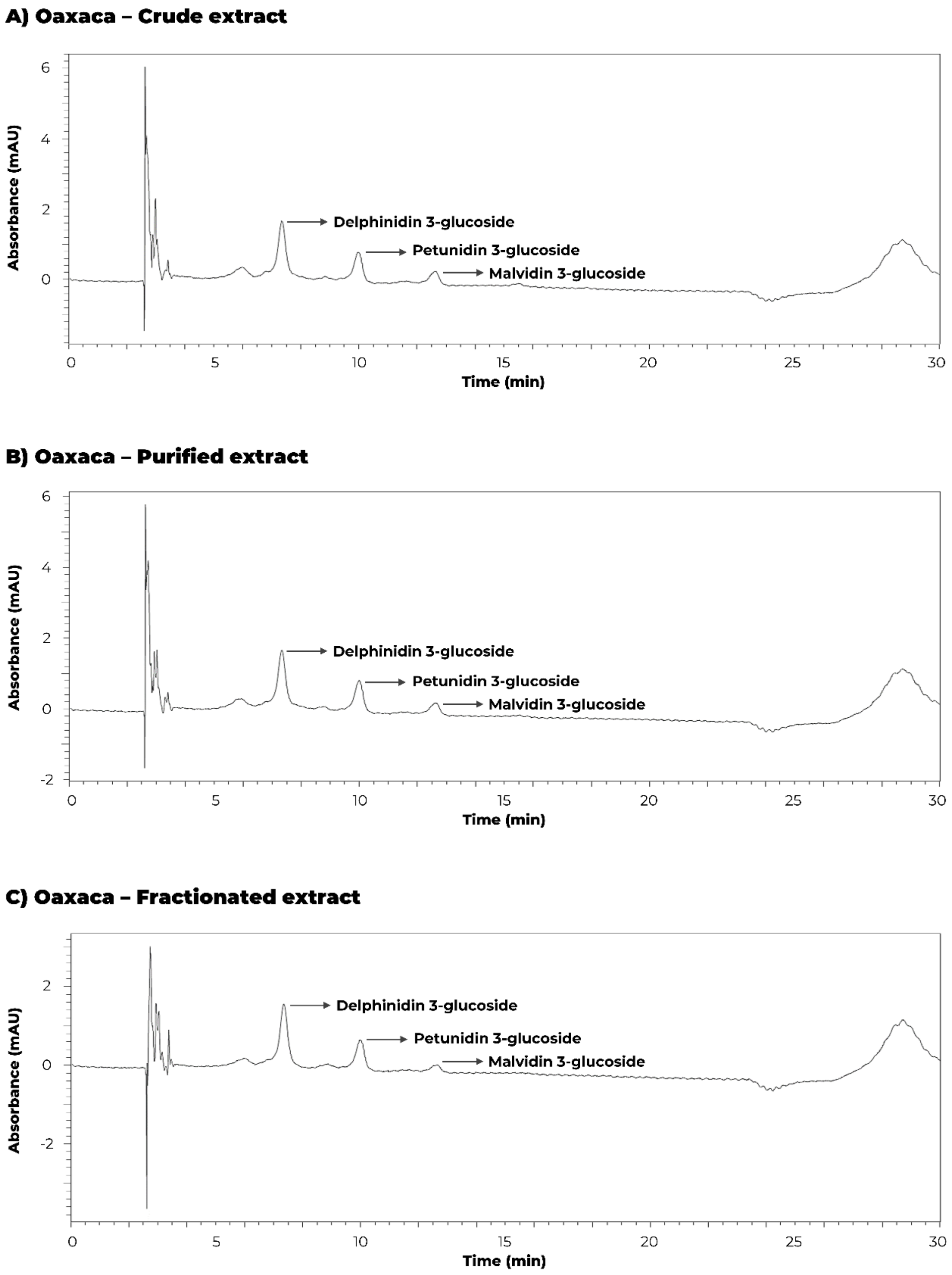

Figure S1. HPLC chromatogram of anthocyanin extracts from Oaxaca black beans. A) Crude anthocyanin-rich extract; B) Purified anthocyanin-rich extract; C) Fractionated anthocyanin-rich extract.

A) Guerrero – Crude extract

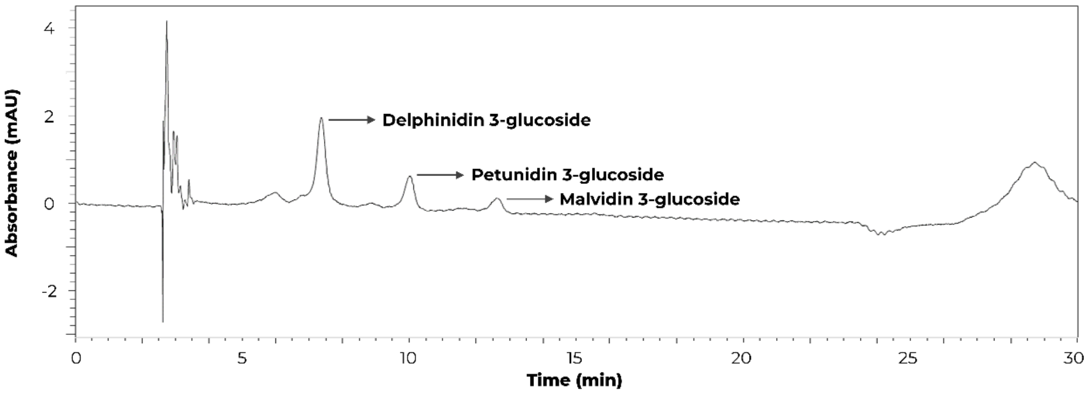

B) Guerrero – Purified extract

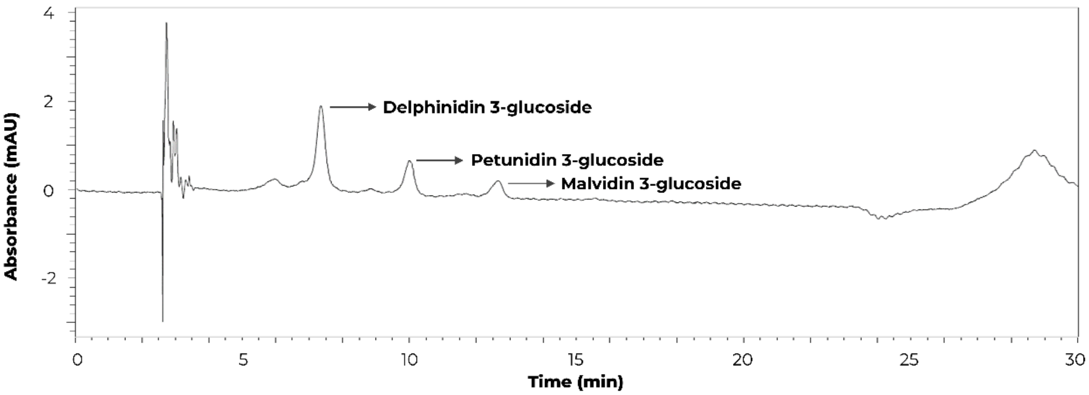

C) Guerrero – Fractionated extract

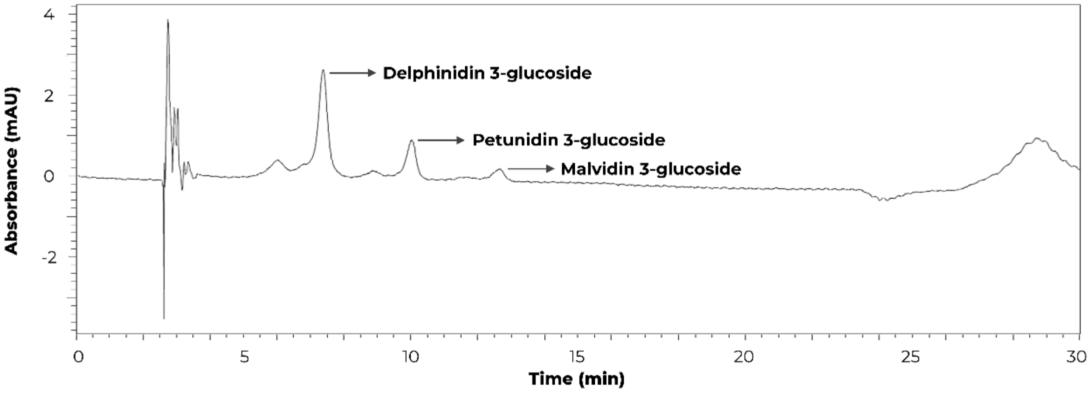

Figure S2. HPLC chromatogram of anthocyanin extracts from Guerrero black beans. A) Crude anthocyanin-rich extract; B) Purified anthocyanin-rich extract; C) Fractionated anthocyanin-rich extract.

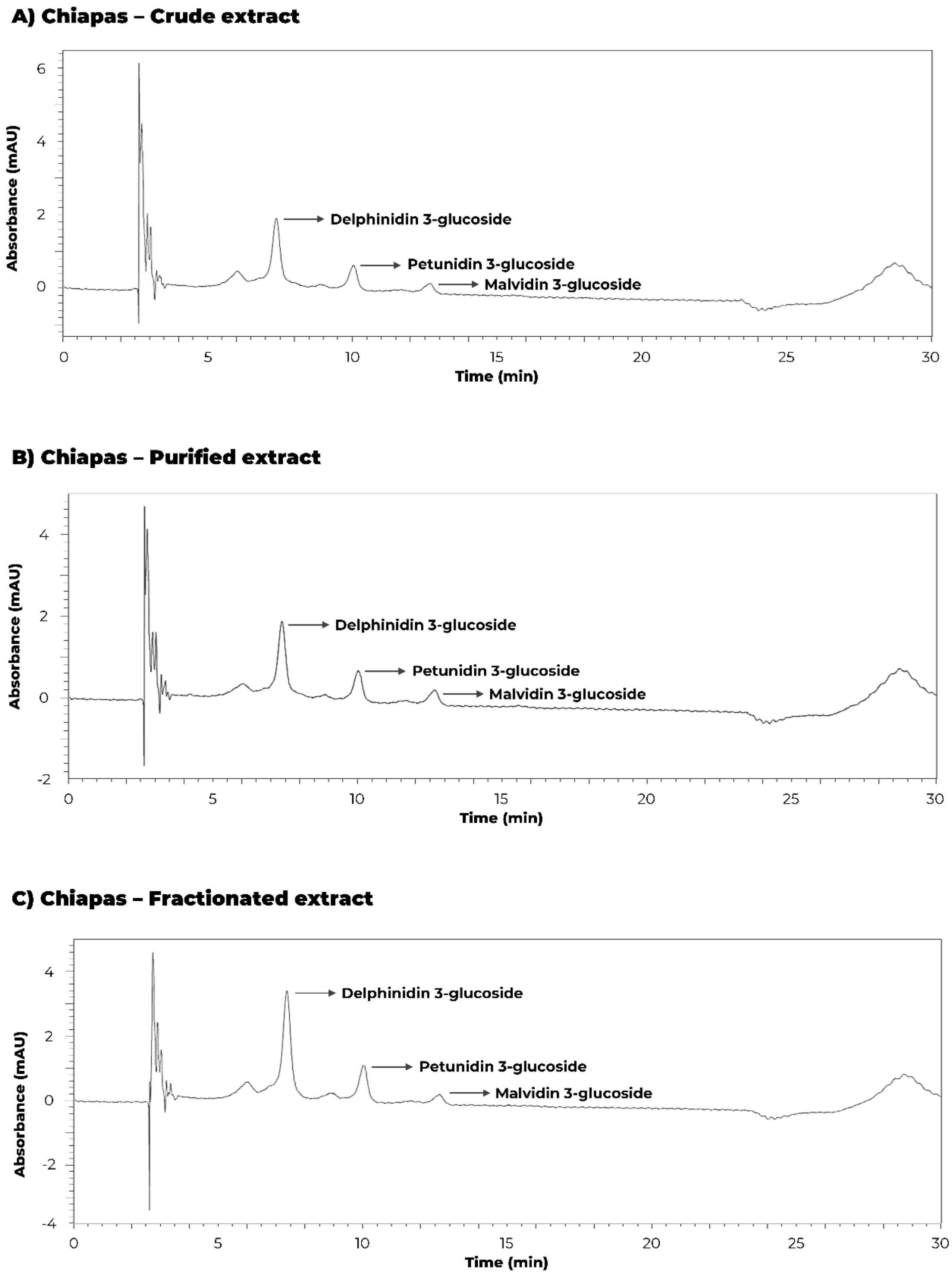

Figure S3. HPLC chromatogram of anthocyanin extracts from Chiapas black beans. A) Crude anthocyanin-rich extract; B) Purified anthocyanin-rich extract; C) Fractionated anthocyanin-rich extract.

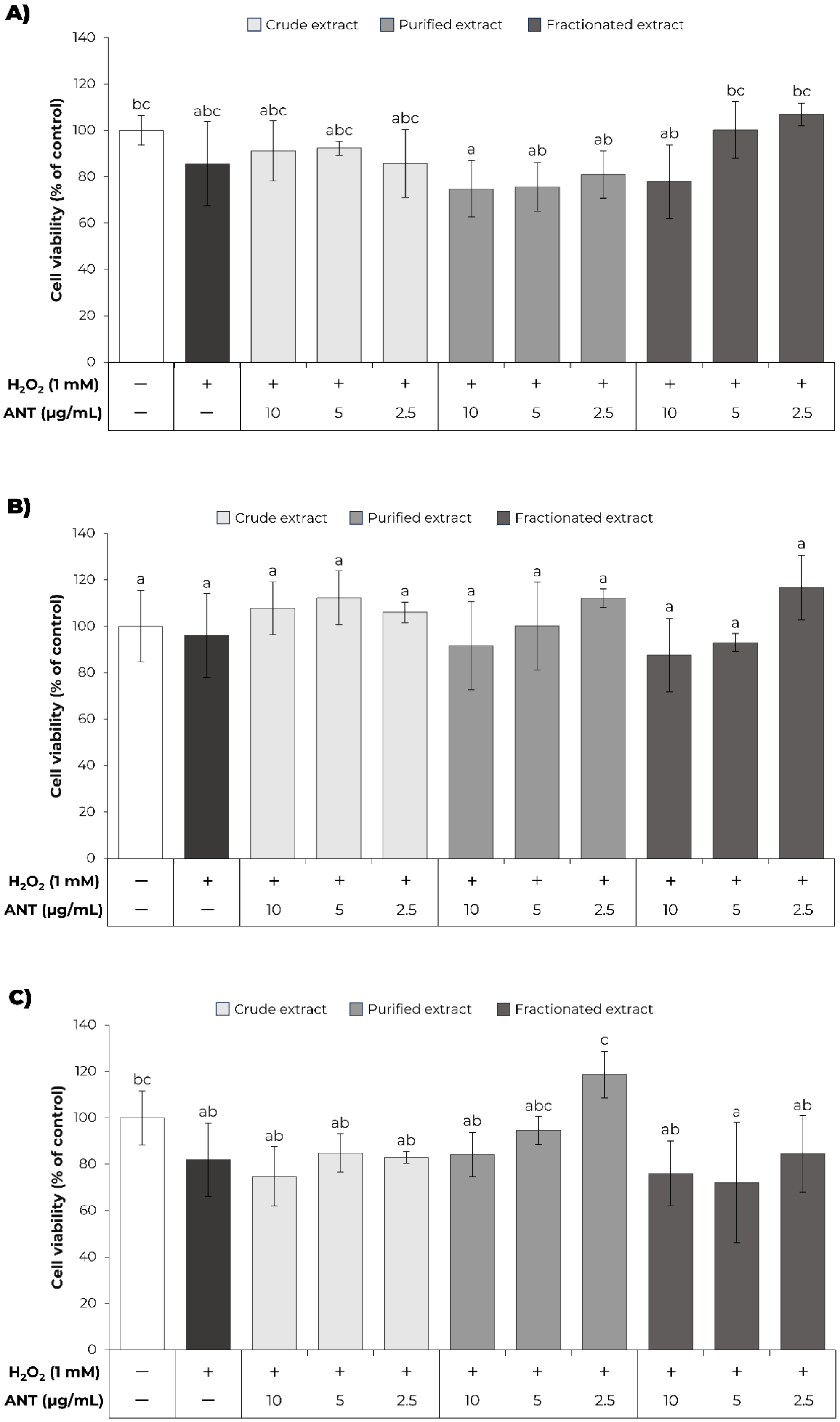

Figure S4. Effect of anthocyanin extracts on cell viability in RAW 264.7 macrophages stimulated with 1 mM hydrogen peroxide (H_2O_2). A) Oaxaca black beans; B) Guerrero black beans; C) Chiapas black beans. Mean values ± SD. Values with different letters indicate significant ($p < 0.05$) differences among treatments using ANOVA and Tukey's post hoc test.
